# Relationship between regional volume changes and water diffusion in fixed marmoset brains: an in vivo and ex vivo comparison

**DOI:** 10.1038/s41598-024-78246-0

**Published:** 2024-11-06

**Authors:** Daisuke Yoshimaru, Tomokazu Tsurugizawa, Naoya Hayashi, Junichi Hata, Shuhei Shibukawa, Kei Hagiya, Hinako Oshiro, Noriyuki Kishi, Kazuhiro Saito, Hideyuki Okano, Hirotaka James Okano

**Affiliations:** 1 Division of Regenerative Medicine, The Jikei University School of Medicine, 3-25-8, Nishi-shinbashi, Minato-ku, Tokyo, 105-8461 Japan; 2 Laboratory for Marmoset Neural Architecture, RIKEN Center for Brain Science, Saitama, Japan; 3 National Institute of Advanced Industrial Science and Technology (AIST), Tsukuba, Japan; 4 Faculty of Engineering, University of Tsukuba, Tsukuba, Ibaraki Japan; 5 Graduate School of Human Health Sciences, Tokyo Metropolitan University, Tokyo, Japan; 6 Department of Radiology, Tokyo Medical University, Tokyo, Japan; 7 Department of Physiology, Keio University School of Medicine, 35 Shinanomachi, Shinjuku-ku, Tokyo, 160-8582 Japan; 8 Faculty of Health Science, Department of Radiological Technology, Juntendo University, Tokyo, Japan

**Keywords:** Translational research, Neural ageing

## Abstract

Ex vivo studies of the brain are often employed as experimental systems in neuroscience. In general, brains for ex vivo MRI studies are usually fixed with paraformaldehyde to preserve molecular structure and prevent tissue destruction during long-term storage. As a result, fixing brain tissue causes microstructural changes and a decrease in brain volume. Therefore, the purpose of this study was to investigate the regional effect of brain volume and microstructural changes on the restricted diffusion of water molecules in the common marmoset brain using in vivo and ex vivo brains from the same individual. We used 9.4T magnetic resonance imaging and also compared the T2-weighted images and diffusion-weighted imaging (DWI) data between in vivo and ex vivo brains to investigate changes in brain volume and diffusion of water molecules in 12 common marmosets. We compared fractional anisotropy, mean diffusivity, AD (axial diffusivity), and radial diffusivity values in white matter and gray matter between in vivo and ex vivo brains. We observed that AD showed the strongest correlation with regional volume changes in gray matter. The results showed a strong correlation between AD and changes in brain volume. By comparing the in vivo and ex vivo brains of the same individual, we identified significant correlations between the local effects of perfusion fixation on microstructural and volumetric changes of the brain and alterations in the restricted diffusion of water molecules within the brain. These findings provide valuable insights into the complex relationships between tissue fixation, brain structure, and water diffusion properties in the marmoset brain.

**Supplementary Information:**

The online version contains supplementary material available at 10.1038/s41598-024-78246-0.

## Introduction

Magnetic resonance imaging (MRI) is an established tool to assess the brain phenotype based on both function and morphology^1–3^. Regional volume in white matter and gray matter reflects the density of neurons, glial cells, or myelin^4^. These factors are known to change with age, and consequently, regional brain volume is associated with aging^5,6^. In addition to volumetry, diffusion-weighted imaging (DWI), which detects the restricted diffusion of water molecule in the intra/extracellular space, is sensitive to brain microstructure^7^. Restricted diffusion refers to the restriction of water molecule movement by physical barriers such as cell membranes or by interactions within a tight environment such as cell growth. The degree of this restriction changes the DWI signal. Therefore, DWI is altered by neurological diseases that induce cell loss^8,9^. In addition, DWI is impacted by cell growth as in cytotoxic edema. The relationship between cellular volume change and water molecule diffusion is demonstrated using the Aplysia ganglion^10,11^. Volume change of neurons and astrocytes may alter water diffusion in rodent brains^12–14^. The diffusion coefficient and anisotropy of water molecule diffusion may be affected by changes in cellular membranes^15^. These reports suggest that water diffusion may be affected by changes in brain microstructure.

For ex vivo MRI studies, brains are usually fixed with paraformaldehyde to preserve the molecular structure and prevent tissue destruction during long-term storage^16,17^. Ex vivo studies of the brain are often employed as experimental systems in neuroscience. However, the fixing brain tissue causes microstructural changes and a reduction in brain volume^17,18^. In addition, it has been reported that the diffusion anisotropy in mouse brain is altered after perfusion fixation^19^.

Therefore, in this study, we aimed to investigate the relationship between regional brain volume changes, microstructural alterations, and restricted diffusion of water molecule in the brain. In order to assess the effects solely attributed to changes in brain volume and microstructure, while excluding influences due to inter-individual background differences, both in vivo and ex vivo brains from the same subject were utilized. In this study, common marmosets (Callithrix jacchus) were employed due to their brain structures being more similar to humans than those of rodent, making them valuable for researching higher cognitive brain functions and human brain diseases, which are not adequately modeled in rodents.

## Results

**Fig. 1:**
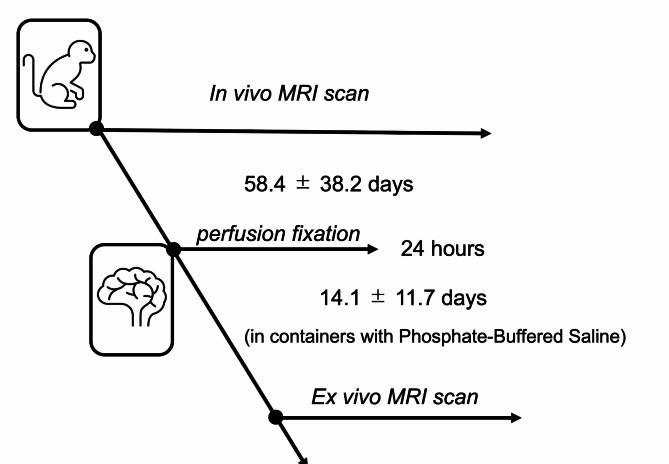
Schematic of experimental paradigm from in vivo brain imaging to ex vivo brain imaging of common marmosets. Experimental schedule from the in vivo MRI scan to the ex vivo MRI scan after perfusion fixation.

T2-weighted and DWI data were obtained in ex vivo and in vivo MRI to investigate changes in brain volume and water molecule diffusion. The experimental schedule from in vivo MRI scan to ex vivo MRI scan is shown in Fig. 1.

### Regional volume reductions in ex vivo brains

**Fig. 2:**
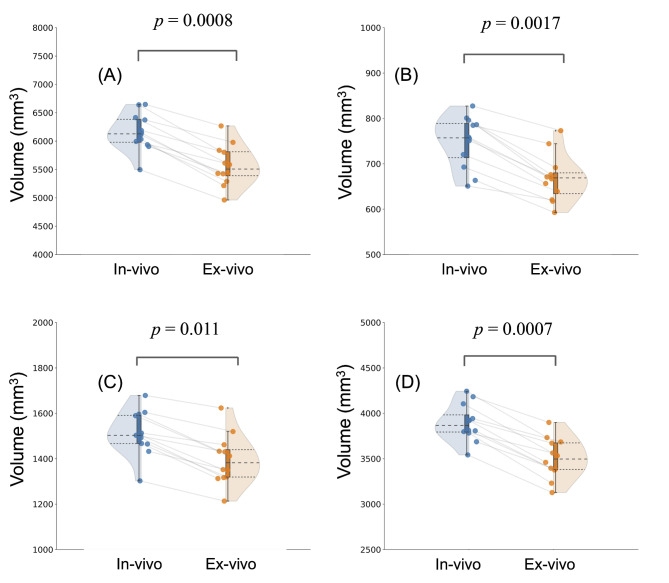
Comparison of brain volumes between in vivo and ex vivo brains. Comparison of whole brain (**A**), cerebellum (**B**), white matter (**C**), and gray matter (**D**) volumes between in vivo and ex vivo brains. Statistical results are shown in the upper part of the graphs. Statistical analysis was performed with a Bonferroni-corrected paired t-test (α = 0.05).

**Fig. 3:**
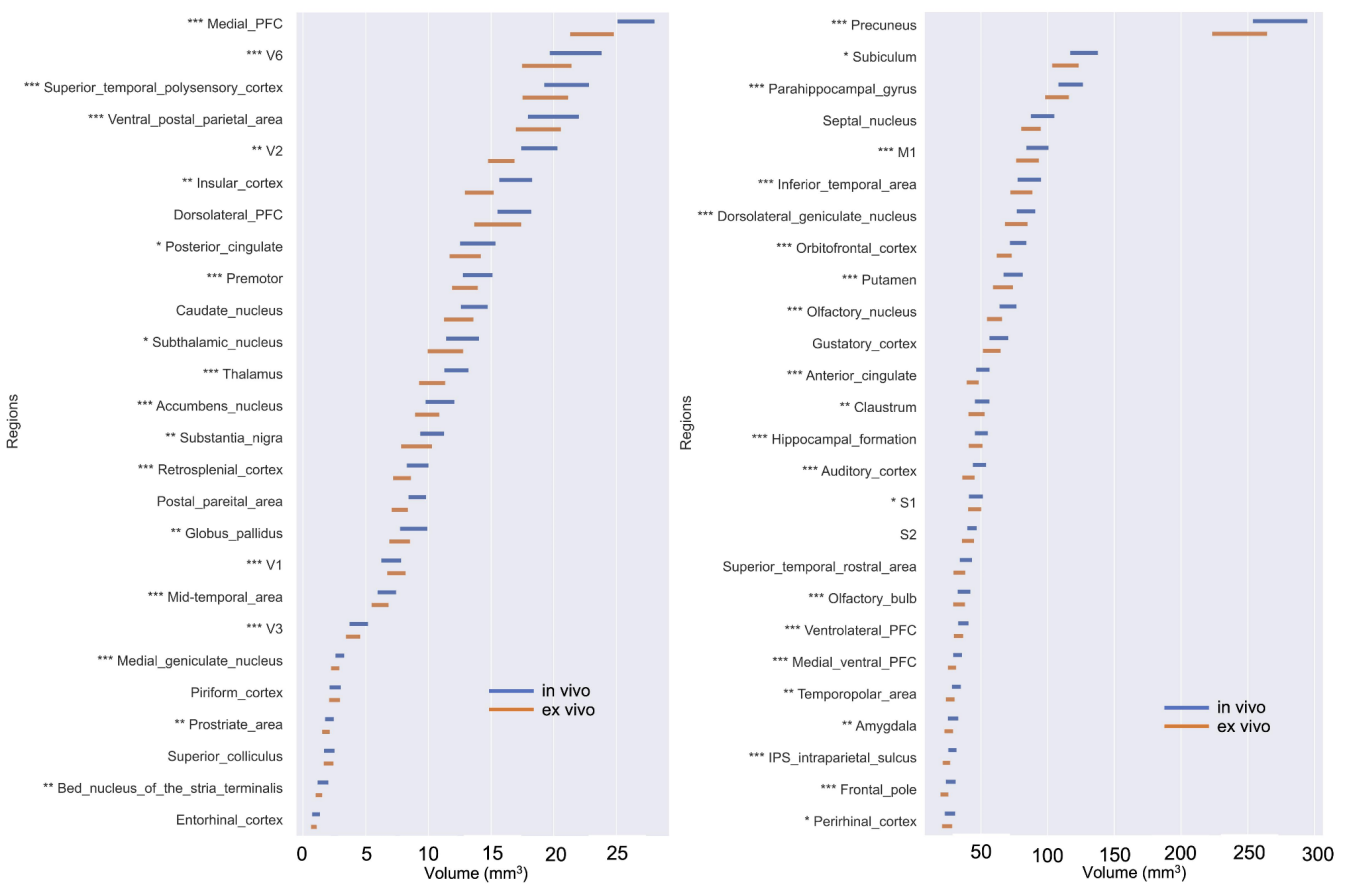
Comparison of volume changes in 52 whole-brain regions between in vivo and ex vivo brains. The figures are arranged in order of increasing brain volume and divided into two parts. The length of the bars in each region indicates the width of the standard deviation. In vivo and ex vivo statistical results are indicated by an asterisk to the upper left of the region name (*p < 0.05, **p < 0.01, ***p < 0.0001). Statistical analysis was performed with a Bonferroni-corrected paired t-test.

In vivo and ex vivo volume comparisons of the whole brain, cerebellum, white matter, and gray matter are shown in Fig. 2. The ex vivo brain volume was significantly reduced in all four regions compared to the in vivo brain volume. The changes in white matter were 0.86 ± 0.24, in gray matter were 0.84 ± 0.23, in the cerebellum were 0.83 ± 0.23, and in the whole brain were 0.84 ± 0.24. These reductions were homogeneous across the different brain regions. Furthermore, we averaged the volumes of the same brain regions on the left and right sides, and no significant differences were observed between them. This approach was chosen to focus on overall structural changes due to perfusion fixation, rather than potential hemispheric differences, which were not the primary interest of this study. We examined the volume changes in each of these 52 regions (Fig. 3). The volumes of each brain region were compared between in vivo and ex vivo brains. The graphs are ordered by volume from top to bottom. There was a large decrease in volume after perfusion fixation. Overall, there was a decreasing trend in volume with perfusion fixation; however, there were no statistically significant differences between in vivo and ex vivo volumes in the following 10 regions: the piriform cortex, entorhinal cortex, dorsal lateral prefrontal cortex, septal nucleus, gustatory cortex, secondary somatosensory cortex, superior temporal rostral area, posterior parietal area, caudate nucleus, and superior colliculus (Supplementary Fig. 1).

### Diffusion index change correlated with volume reduction

**Fig. 4:**
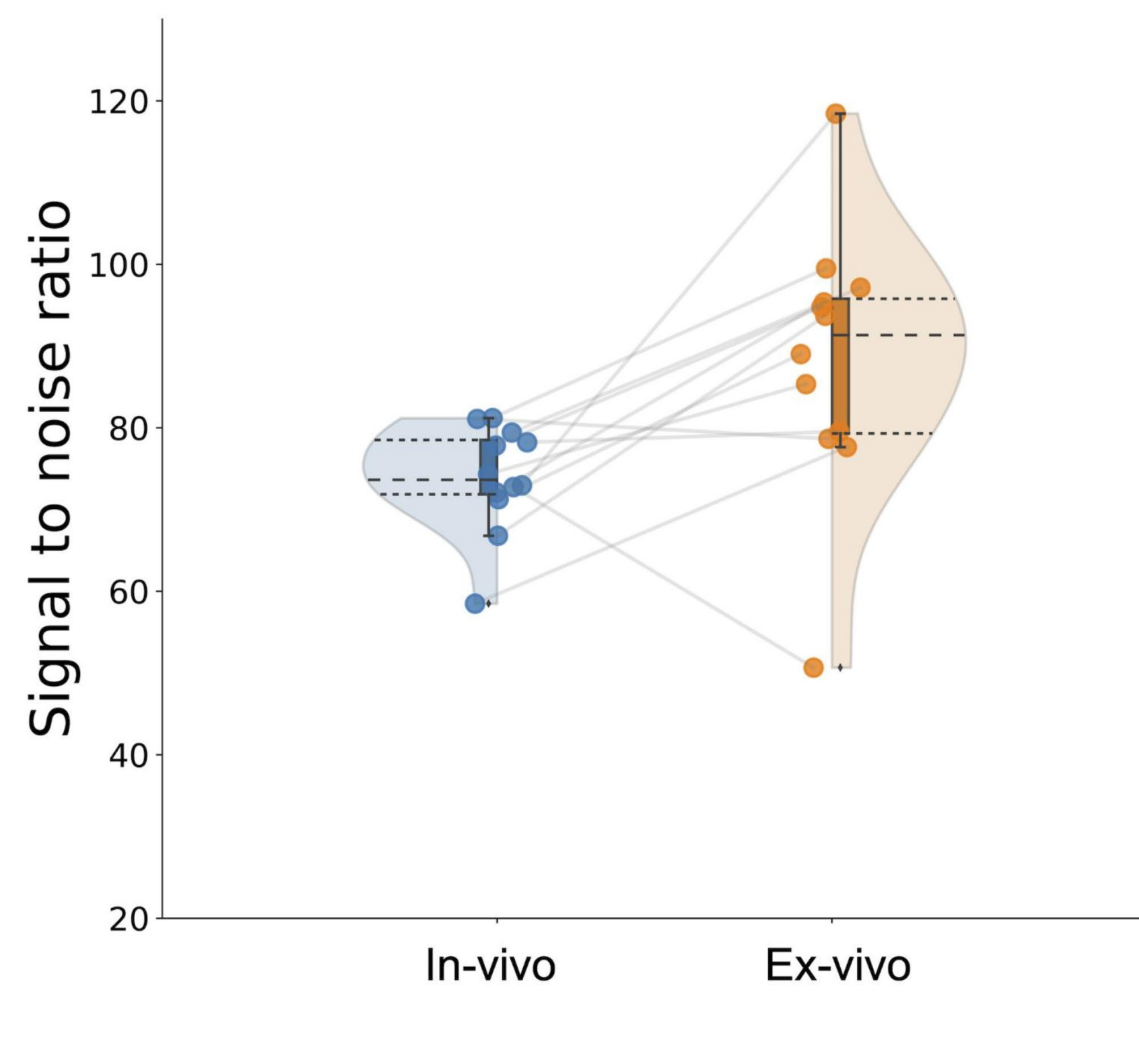
SNR of each in vivo and ex vivo DWI data. SNR was measured using QIBA (Quantitative Imaging Biomarker Alliance) in 2019 according to the methods presented in the “QIBA Diffusion Profile”.

To ensure the reproducibility of the diffusion tensor indices, we measured the signal-to-noise ratio (SNR) of the DWI data. The mean SNR in DWI data was 73.9 ± 6.5 for in vivo and 88.3 ± 16.4 for ex vivo (Fig. 4). Furthermore, the actual signal values used for the calculations are shown in the Supplemental Table 1.

We then compared fractional anisotropy (FA) values, mean diffusivity (MD), axial diffusivity (AD), and radial diffusivity (RD) values in white matter and gray matter in vivo and ex vivo brains (Table 1). The FA value in white matter was significantly increased in the ex vivo brain. However, the FA value in the gray matter of the ex vivo brain was not significantly different from that of in vivo brain. Furthermore, in AD, MD, and RD, the values of each index were significantly reduced in the white and gray matter of the ex vivo brain.

**Table 1:** Comparison of each diffusion index between* in vivo* and* ex vivo* brain.

DTI index	Region	*In vivo*	*Ex vivo*	Effect size	*p* value	Significance
FA	White matter	0.48 ± 0.02	0.53 ± 0.02	0.39	<0.01	**
	Gray matter	0.23 ± 0.01	0.23 ± 0.01	0.06	0.71	
AD (×10^−4^ mm^2^/s)	White matter	11.1 ± 1.0	5.6 ± 0.9	0.82	<0.01	**
	Gray matter	8.5 ± 0.5	6.0 ± 0.6	0.75	<0.01	**
MD (×10^−4^ mm^2^/s)	White matter	6.3 ± 1.5	2.7 ± 1.3	0.77	<0.01	**
	Gray matter	6.4 ± 1.2	3.9 ± 1.2	0.76	<0.01	**
RD (×10^−4^ mm^2^/s)	White matter	4.4 ± 1.3	1.6 ± 1.0	0.79	<0.01	**
	Gray matter	5.5 ± 1.3	3.0 ± 1.3	0.85	<0.01	**

* p < 0.05, ** p < 0.01 (all p-values are Bonferroni-corrected).

**Fig. 5:**
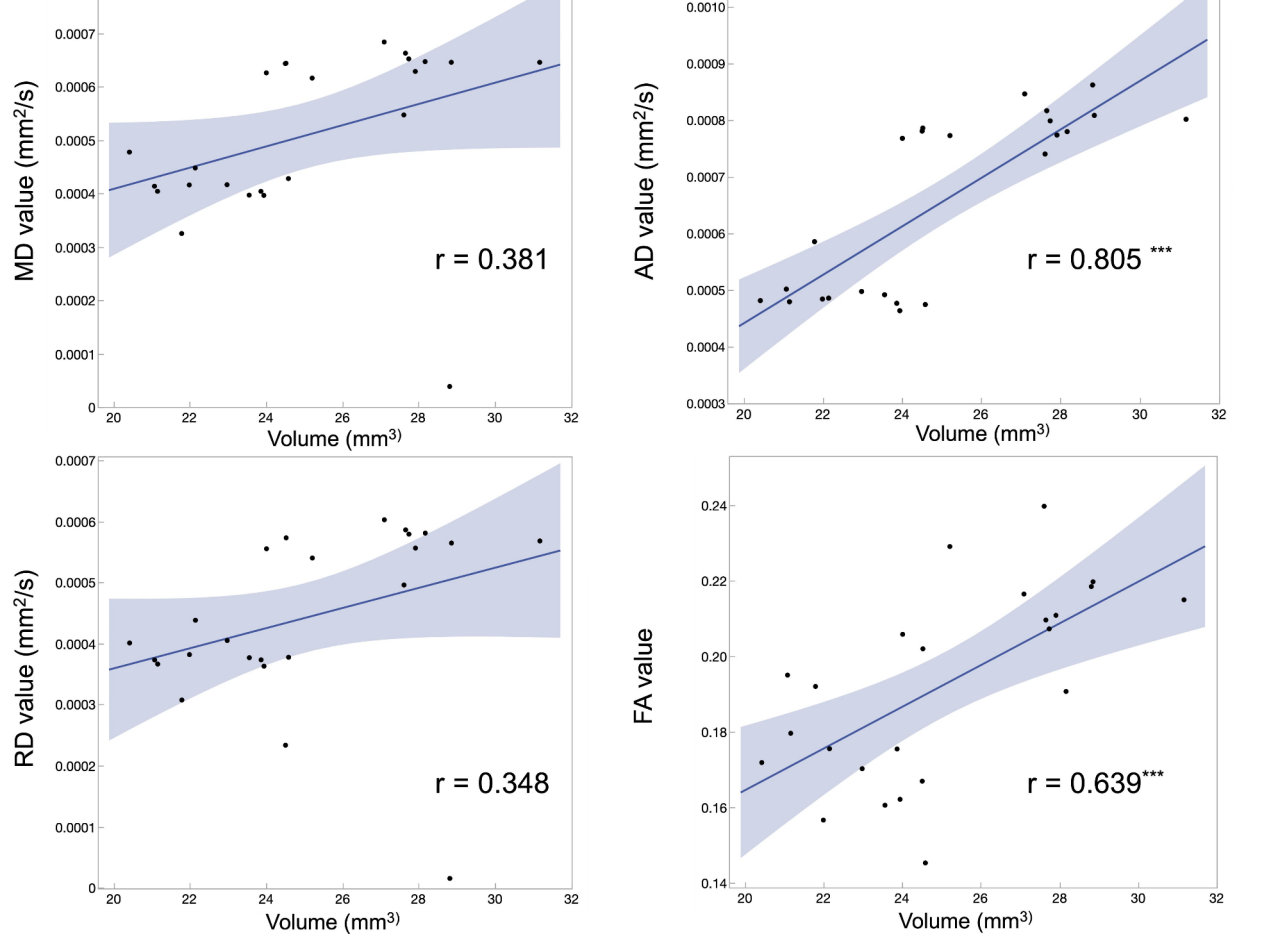
The relationship between DTI indices and volume changes in brain regions. The figure shows the correlation between the DTI indices MD, AD, RD, and FA and the volume change of brain regions. The results for the frontal pole are shown here as an example. The Pearson correlation coefficient (r) was used to assess the linear relationship between brain volume and DTI indices. The upper and lower lines of the correlation line indicate 95% confidence intervals. Significant correlations are indicated by an asterisk in the upper right corner of the correlation coefficient (*p < 0.05, **p < 0.01, ***p < 0.0001).

In addition, we investigated the correlation of DTI (Diffusion Tensor Imaging) indices (FA, MD, AD, and RD) with volume changes of white matter, gray matter, and 52 brain regions. White matter volume change was significantly correlated with AD and FA, whereas gray matter volume change showed significant correlations with MD and AD (Table 2). FA showed a significant correlation between the DTI indices and volume change in 24 of the 52 regions (Supplementary Table 2). Of note, most of these correlations were negative. In addition, MD and RD showed significant correlations with nine and six regions, respectively (Supplementary Tables 3 and 4). For each DTI index, AD was correlated with volume change in 40 brain regions (Supplementary Table 5). Moreover, its correlation coefficient was higher than that of all the other DTI indices. As an example of the relationship between each DTI index and brain region volume change, the results for the frontal pole are shown in Fig. 5. Similarly, to investigate changes between individuals, volume and diffusion indices were subtracted between in vivo and ex vivo, and correlations between these volume and diffusion index differences in the white matter and each brain region are shown in Supplementary Tables 6–10.

**Table 2:** Correlation of DTI indices with volume changes of white matter and gray matter.

DTI index	Region	*r*	*p* value	Significance
FA	White matter	−0.49	0.0161	*
	Gray matter	0.03	0.8724	
AD	White matter	0.57	0.0038	**
	Gray matter	0.66	0.0004	***
MD	White matter	0.28	0.1861	
	Gray matter	0.42	0.0404	*
RD	White matter	0.23	0.28	
	Gray matter	0.39	0.0622	

* p < 0.05, ** p < 0.01, *** p < 0.001 (all p-values are Bonferroni-corrected).

## Discussion

In this study, using the brain of the common marmoset, we elucidated the effects of perfusion fixation on microstructural and volumetric changes and their impact on diffusion metrics of water molecule within the brain. Previous studies have seldom precisely investigated the changes accompanying the transition from in vivo to ex vivo brains. Since the in vivo and ex vivo models are not based on the same individuals, the results include inter-individual structural variability. In contrast, our study successfully conducted intergroup comparison between the same individual’s in vivo and ex vivo brains by examining the volume and diffusion metrics. This approach allowed us to adequately demonstrate the relationship between volume and diffusion metrics in the same brain under in vivo and ex vivo conditions. Furthermore, our analysis revealed that AD exhibited the strongest correlation with local volume changes among the diffusion metrics examined.

## Diffusion indices and regional volume

The present study shows the difference in diffusion indices between in vivo and ex vivo brains. However, it can be argued that this difference is due to the SNR of the scanning parameters between in vivo and ex vivo, such as b values, repetition time, and spatial resolution. In general, the SNR at b = 0 s/mm^2^ is recommended to exceed 50 ± 5 for the reproducibility of DTI indices^20^. The observed variability in SNR can be attributed to two main factors. Firstly, the limited number of specimens used in our study may have contributed to this variability. Additionally, a significant factor for ex vivo imaging was the inconsistency in the environmental conditions within the imaging container, such as the presence of minute air particles, compared to in vivo brain imaging. The presence of air within the ventricles could also potentially lead to a decrease in signal intensity. Despite conducting the imaging process with care, these factors could have contributed to slight inconsistencies in the signal observed. The SNR of the DWI data used in this study exceeded 50 for both in vivo and ex vivo data, indicating sufficient SNR of the DWI data. In this study, the number of gradient directions for diffusion differs between in vivo and ex vivo. However, it has been previously reported that there is no difference in the eigenvectors representing diffusion anisotropy when more than 20 axes are used^21–23^. As the minimum number of axes we utilized is 32, we consider the impact on DTI indices to be minimal.

Similarly, due to differences in spatial resolution, we acknowledge the potential influence of partial volume effects. Nevertheless, previous studies have shown that differences in spatial resolution do not significantly alter the FA of white matter, reporting a decrease in variability with higher resolution^24^. Moreover, it is known that higher resolution can reduce the effects of partial volume^25^. These findings were gathered from human brain data collection, where the spatial resolution is around 2 mm. Our investigation employed a thickness of 0.7 mm and an in-plane resolution of 0.35 mm, which are considerably smaller. Given reports that the size of neuronal cell bodies in primates, such as humans and marmosets^26^, are relatively similar, we do believe there is an influence of partial volume effects, but it is considered to be minimal.

A more detailed consideration of the impact of temperature on diffusion is necessary. Richardson et al. compared viable and fixed tissues at physiological temperature (37 °C), demonstrating that differences in diffusion characteristics are primarily due to changes in tissue microstructure^27^.

Their study revealed that the fixation process leads to a decrease in AD and an increase in RD. Our findings show a similar trend, albeit with a slight difference in magnitude.

In our study, we observed a strong positive correlation between AD and volume changes, which aligns well with Richardson et al.‘s finding of decreased AD with fixation. Regarding RD, while Richardson et al. reported an increase, our results showed a weaker correlation with volume changes. These changes in AD and RD show some similarities with previous findings, although there are differences.

Furthermore, Oshiro et al. showed that for small structural scales (6–12 μm) and specific diffusion times (over 50ms), there is minimal change in the diffusion coefficient due to temperature. The diffusion times used in our study (ex vivo: 11.667ms, in vivo: 10ms) fall within this range^28^. Considering the size of marmoset brain cells (0.5–50 μm)^29^, the direct impact of temperature may be minimal in many regions.

These findings suggest that the differences we observed between in vivo and ex vivo conditions are primarily attributable to microstructural changes in the tissue due to the fixation process, with the direct impact of temperature being secondary. However, a more detailed examination of temperature effects, particularly on water molecule diffusion in extracellular spaces, is warranted.

When comparing diffusion indices between white and gray matter, significant reductions in AD, MD, and RD were observed in the ex vivo brain compared to the in vivo brain (Table 1). AD, RD, and MD changes in gray matter are interpreted as changes in tissue integrity and extracellular space^30^, whereas AD, RD, and MD changes in white matter indicate myelin changes^31^. These results indicate that the reduction of water molecule diffusion in the ex vivo brain is not tissue-dependent and in all directions. In contrast, FA was significantly increased in the white matter of the ex vivo brain but was unchanged in the gray matter compared to the ex vivo brain. The FA increase is related to the volume change of the white matter (Table 2). This is because FA is the directional selectivity of the random diffusion of water molecule within the myelinated bundles that form the white matter^31^. It has been reported that FA changes with fixation in the white matter of mice^32^. Another study reported that the FA values of a fixed white matter were unchanged with fixation; however, AD, RD, and MD were changed with fixation^33^. This may be because they were compared diffusion metrics of in vivo and ex vivo brains in different animals were compared, and individual differences in FA were taken into account. Furthermore, this study demonstrated that, in intergroup comparisons within the same subjects, white matter FA changed after fixation. In addition, because gray matter has fewer anisotropic water molecule than white matter, fixation-related FA changes would have been smaller in gray matter than in white matter.

Notably, AD showed a significant correlation with volume change in the greatest number of regions (40 out of 52 total brain regions) compared to RD, MD, and FA. AD and RD represent the diffusion coefficients in the long- and short-axis directions, respectively, while MD is the mean diffusion of all directions. Our results suggest that AD may be the most sensitive to tissue volume change, as it showed stronger correlations than MD, which averages diffusion across all directions. These findings indicate that volume changes may have significantly contributed to the observed alterations in AD.

## Perfusion and regional volume change

Perfusion fixation significantly reduced the volume of the whole brain, white matter, gray matter, and cerebellum because the formalin fixative solution caused a gradual contraction process due to crosslink formation and soluble solid dissolution^34^. Furthermore, de Guzman et al. reported nonuniform volume changes in the mouse brain and nonsignificant shrinkage in multiple regions, which is a consideration of the different composition of each cell^18^. The current study showed that the brain areas, including several regions, such as the entorhinal cortex, dorsal lateral prefrontal cortex, and secondary somatosensory cortex, had non-significant volume changes between in vivo and ex vivo brains. These regions are located relatively far from the center of the brain and are dominated by peripheral blood flow^35,36^. It is possible that fixation in the center of the brain was inadequate compared to surface areas where blood flow is controlled by perfusion. Furthermore, because the marmoset brain has fewer sulci, the effect of fixation from a fixative solution located outside of the brain may also be significantly less than that at the surface. This suggests that, in contrast to small mouse brains, the nonuniform volume change due to the fixation in the marmoset brain is due not only due to the cellular composition but also due to the influence of regional perfusion.

## Limitation of study

This study has several limitations. First, the coils used in this study were different for in vivo and ex vivo experiments; therefore, the imaging parameters were not the same. However, since sufficient spatial resolution and SNR were ensured, we believe that the effect on volume calculation and diffusion indices is minimal. Second, there is a temperature difference between in vivo and ex vivo measurements. While previous studies suggest that the impact of temperature on diffusion in restricted environments may be minimal for certain structural scales and diffusion times, we cannot completely rule out its influence. However, our analysis demonstrates that the observed differences between in vivo and ex vivo conditions are primarily attributable to microstructural changes in the tissue resulting from the fixation process. Ideally, ex vivo measurements at physiological temperature would help to further clarify the potential impact of temperature differences. However, we were unable to perform such measurements in this study due to the unavailability of the original ex vivo samples for additional MRI scanning.

It’s important to note that while temperature may have some effect, particularly on water molecule diffusion in extracellular spaces, the restricted diffusion environment within biological tissues, especially in fixed samples, likely reduces the temperature dependency of water molecule movement. Finally, this study primarily focused on the relationship between brain volume changes and diffusion metrics following fixation. However, several limitations must be acknowledged:

1. Fixation-induced microstructural changes: Fixation induces microstructural alterations beyond volume changes, such as modifications in water exchange rates between intra- and extracellular spaces. These additional changes may contribute to the observed differences in diffusion properties.
2. Time gap between in vivo scanning and fixation: A significant time interval existed between in vivo scanning and tissue fixation, during which the animals were used for other experiments. This delay potentially allowed for structural brain changes due to normal plasticity or experimental interventions.

Given these limitations, relationship between volume changes and diffusivity alterations can be not only due to the fixation process, but also other intervening factors, including unquantified microstructural changes and potential brain plasticity during the inter-scan period. While our findings provide valuable insights into the relationship between volume changes and diffusion properties in fixed marmoset brains, they should be interpreted cautiously.

## Conclusions

In this study, we showed that AD is most sensitive to regional gray matter volume. By comparing the in vivo and ex vivo brains of the same individual, we identified significant correlations between the local effects of perfusion fixation on microstructural and volumetric changes of the brain and alterations in the restricted diffusion of water molecule within the brain. These findings provide valuable insights into the complex relationships between tissue fixation, brain structure, and water diffusion properties in the marmoset brain.

## Materials and methods

### Animals and image acquisition

This study was approved by the Animal Experiment Committees at the RIKEN Brain Science Institute and conducted per the Guidelines for Conducting Animal Experiments of the RIKEN Brain Science Institute (H27-2-307). All methods are reported in accordance with ARRIVE guidelines (https://arriveguidelines.org ). Common marmosets (*n* = 12; 6.2 ± 2.1 years; 1 males and 11 females) were used in this study. MRI was performed using a 9.4T BioSpec 94/30 (Biospin GmbH, Ettlingen, Germany). A transmitting and receiving volume coil with an 86-mm inner diameter was used for in vivo brains, and a transmitting and receiving solenoid type coil with a 28-mm inner diameter was used for ex vivo brains. T2-weighted images and DWI data were obtained to investigate changes in brain volume and water molecule diffusion in these subjects.

### In vivo MRI

T2-weighted images were acquired using a rapid acquisition with refocused echoes (RARE) sequence with the following parameters; TR = 4000 ms; TE = 22 ms, RARE factor = 4; field of view = 48 × 48 mm^2^, matrix = 178 × 178, and acquisition time = 7.4 min. DWI data were acquired using spin-echo echo-planar-imaging sequences with the following parameters; repetition time = 3,000 ms, echo time = 25.6 ms, b value = 1000 s/mm^2^, gradient directions = 32 directions, field of view = 44.8 × 44.8 mm^2^, matrix = 128 × 128, thickness = 0.7 mm, total scan time = 28.8 min from each animal. The diffusion gradient directions were distributed uniformly over a full sphere, which allows for optimal correction of eddy current distortions during the data processing stage. To estimate the inhomogeneous magnetic field, an additional image was acquired with b = 0, exactly in the opposite phase-encoding direction (but with the same readout time). This opposite phase encoding refers to changing the direction of the magnetic field gradient of the phase encoding gradient^37^. The animals were scanned in a supine position on an imaging stretcher and administered a mixture of carrier gas and isoflurane (1.5–2.5% concentration; Abbott Laboratories, Abbott Park, IL, USA) for anesthesia. The carrier gas consisted of a 1:1 mixture of 100% oxygen and room air, resulting in an effective oxygen concentration of approximately 60% in the final mixed gas. This anesthesia protocol was chosen to maintain adequate oxygenation while minimizing the risk of atelectasis, as recommended in previous studies^38,39^. The isoflurane concentration was fine-tuned based on anesthesia depth to maintain an appropriate level of sedation throughout the scanning procedure. During the scan, each animal’s heart rate, peripheral oxygen saturation (SpO2), respiration, and rectal temperature were regularly monitored to manage the animal’s physical condition. The target ranges for these physiological parameters were as follows: heart rate, 140–250 beats/min (upper limit constrained by the maximum detection capability of the heart rate monitor); oxygen saturation, 95–100%; respiratory rate, 20–50 breaths/min; and body temperature, 36–38 °C. All animals maintained these physiological parameters within the target ranges during the experiments, with the possible exception of heart rate, which may have exceeded the monitor’s upper limit of 250 beats/min in some cases, given the naturally high heart rates observed in marmosets (up to 348 beats/min in restrained conditions, as reported in previous studies^40^). Heart rate and SpO_2_ were continuously measured using the Nellcor™ N-BSJP pulse and heart rate monitor (Medtronic, Minnesota). Rectal temperature measurements were conducted using the fiber optic temperature probe interferometry system (ACS-P1-N-62SC, Opsens, Quebec City, Canada). Animal respiratory monitoring and gating were performed using the MP150 and TSD160A (Biopac Systems, Inc.).

### Ex vivo MRI

The perfusion fixation for ex vivo MRI was performed following in vivo MRI scanning (Fig. 1). The mean time-lapse between in vivo MRI and perfusion fixation was 58.4 ± 38.2 days. Subsequently, the specimens were stored in containers with Phosphate-Buffered Saline (PBS) until ex vivo MRI imaging, a storage duration of 14.1 ± 11.7 days. The marmoset used in this study was also used for other experiments such as reproductive engineering, including egg and sperm retrieval, implantation of manipulated fertilized eggs, and parturition, resulting in a large time difference. In addition, the effect of personnel and equipment utilization also affected this time difference. Pentobarbital is administered intraperitoneally at a dose of approximately 50 mg/kg. Following this, ketamine is injected intramuscularly at a dose of approximately 10 mg/kg^41^. This results in the animal entering a deep anesthetic state. Once it is confirmed that the animal is sufficiently anesthetized, the thoracic and abdominal cavities are opened. The muscles are then dissected along the ribs, the diaphragm is detached from the chest wall, the thorax is peeled away towards the caudal side of the ribs, and the sternum is lifted. After recirculating 200 ml of PBS over a period of 20 min, the transcardial perfusion of the common marmoset was performed by injecting 4% paraformaldehyde (PFA) in 0.1 M phosphate buffer (pH 7.4) from the apex to the left ventricle. At this time, the right atrial appendage was cut off. The marmoset brains were then retrieved from the skull and incubated with PFA at 4 °C for 24 h. The fixed brains were replaced in a Fluorinert (FC-72, 3 M, Japan) during MRI scanning. Vacuum degassing was performed to reduce air bubble-derived artifacts. T2-weighted images were acquired with the following parameters; TR = 4000 ms, TE = 22 ms, RARE factor = 4, field of view = 48 × 48 mm^2^, matrix = 178 × 178, and acquisition time = 7.4 min. DWI data were acquired with the following parameters: repetition time = 4,000 ms, echo time = 28.4 ms, b value = 3,000 s/mm^2^, gradient directions = 60 directions, field of view = 38 × 38 mm^2^, matrix = 190 × 190, thickness = 0.2 mm, and total scan time = 173.3 min. The diffusion gradient directions were distributed uniformly over a full sphere, which allows for optimal correction of eddy current distortions during the data processing stage. An additional image was acquired with b = 0, which is the reverse phase encoding of the DWI data, to estimate the inhomogeneous magnetic field^37^. It has been reported that ex vivo brain diffusion coefficients decrease 2–3-fold compared to those of in vivo brains as temperature and tissue microstructural properties change following perfusion fixation^42,43^. Under in vivo conditions, the extra-axonal water signal attenuates with increasing b values, whereas under ex vivo conditions, the extra-axonal space is speculated to contain immobile water trapped in microcompartments, such as glial cell bodies and vesicles^44^. Therefore, we used a higher b value for ex vivo analysis in this study than the b value used in vivo. Ex vivo samples were scanned at room temperature (approximately 22 °C), although the exact temperature was not continuously monitored during the scanning process.

### Image analysis

#### Volume measurements

Individual marmosets were subjected to a skull stripping process to extract in vivo brain images from the head. Volume measurements of the brain were then calculated in the following two ways:

1. A registration map from a white matter atlas, a gray matter atlas, and a predefined 52 left and right brain structure atlas (Supplementary Table 11)^45^ was used to compare brain images and store the deformations. In this research, a multi-approach methodology was employed for image registration to ensure precise alignment across different datasets. Rigid registration was first applied to correct for any translational and rotational discrepancies while preserving the original scale and shape of the images. This step was followed by affine registration, which accommodated changes in image scale, orientation, and position, thereby addressing differences in size and shape across subjects. Lastly, deformable B-spline SyN registration was utilized to capture and correct for local anatomical variations and subtle deformations between the images. These registration techniques were implemented using the ANTs software package, facilitating a comprehensive and accurate alignment process^46^.
2. The determinants from the deformation fields of each subject were used to calculate the volume of each structure. For volume calculations within each segmented region, ITK-SNAP was employed^47^.

Brain volumes of the whole brain, white matter, cerebral cortex, cerebellum, and 52 whole-brain regions were compared in vivo and ex vivo. To provide a clear visual representation of our image processing and analysis pipeline, we have created a detailed flowchart (Supplementary Fig. 2). This flowchart illustrates the step-by-step procedure from initial image acquisition through skull stripping, registration processes, and final volume comparisons between in vivo and ex vivo brain regions. Representative images of in vivo and ex vivo T2-weighted images are shown in supplementary Figs. 3 and 4.

### DTI analysis

#### Verification of DWI data quality

The SNR of the DWI data was measured to assess the reproducibility of these values. Specifically, to ensure the reproducibility of diffusion indices, the Quantitative Imaging Biomarker Alliance (QIBA) released the QIBA Diffusion Profile in 2019^20^. Using the SNR ratio indicated in this profile as the basic performance level of the scan and as a reference, we measured the SNR of each in vivo and ex vivo on the b = 0 diffusion-weighted image via the methods indicated in this QIBA Diffusion profile. The SNR measurements are as follows:

$$\:SNR\:=\:\frac{Spatial\:mean\:voxel\:value\:on\:signal\:image}{Spatial\:standard\:deviation\:voxel\:value\:in\:background}$$

We selected a background region that contains uniform random noise while avoiding signal gradients and structured noise, such as ghosts or severely modulated zones. ROIs (Region of Interest) were established as circular areas with 80–100 pixels, measured at two points in the background and two points within uniform areas of the brain, using the average values to calculate the SNR.

### DTI indices

First, we performed noise reduction on the obtained DWI data^48^. The FSL software-based topup and eddy tools were used to correct for B0 field inhomogeneities, eddy currents, and intervolume motion^49^. From these corrected DWI data, we calculated the DTI indices. The FA values, MD, AD, and RD in the white matter, gray matter, and parcellated brain regions were calculated using the following equations:

AD = λ_1_.

RD = (λ_2_ + λ_3_)/2.

MD = (λ_1_ + λ_2_ + λ_3_)/3.

$$\:FA = \sqrt{\frac{3}{2}}\sqrt{\frac{{\left({\uplambda\:}1-\text{M}\text{D}\right)}^{2}+{\left({\uplambda\:}2-\text{M}\text{D}\right)}^{2}+{\left({\uplambda\:}3-\text{M}\text{D}\right)}^{2}}{{({\uplambda\:}1\:+\:{\uplambda\:}2\:+\:{\uplambda\:}3)}^{2}}}$$

where λ_1_, λ_2_, λ_3_ are eigenvalues.

In Supplemental Fig. 5, we presented samples of in vivo and ex vivo DWI images. Furthermore, in Supplemental Fig. 6, we showed the FA, MD, AD, and RD images obtained from our analysis.

### Statistical analysis

All statistical analyses were carried out with JMP software (version 14.0 for Macintosh, SAS Inc., Cary, NC, USA). We used paired t-test with Bonferroni correction (α = 0.05) to compare in vivo and ex vivo volume measurements, FA values, MD values, AD values, and RD values. The Bonferroni correction factor was computed separately for each statistical analysis, using the number of tests performed within that specific analysis. The Pearson correlation coefficient (r) was used to assess the linear relationship between brain volume and DTI indices. We assessed normality using the Kolmogorov-Smirnov test.

## Electronic supplementary material

Below is the link to the electronic supplementary material.

Supplementary Material 1

## Data Availability

The data supporting the reported findings are available from the corresponding author upon reasonable request.
