# Supplementary material for: Relationship between regional volume changes and water diffusion in fixed marmoset brains: an in vivo and ex vivo comparison
Source: Sci Rep. 2024 Nov 6;14:26901. doi: 10.1038/s41598-024-78246-0 (PMC11541870; doi:10.1038/s41598-024-78246-0)
Supplement: Supplementary file 1 — Supplementary Material 1 [file 41598_2024_78246_MOESM1_ESM.pdf]

Supplementary Table 1.

	in vivo			ex vivo		
	**Area**	**Signal intensity**	**SD**	**Area**	**Signal intensity**	**SD**
**Background**	**82.93**	**138.72**	**20.88**	**83.18**	**155.34**	**25.93**
**Spatial mean voxel**	**83.77**	**1528.08**	**316.91**	**82.20**	**2290.15**	**660.89**

Supplementary Table 2. Significant correlation between brain region volume and FA values and effect size

**Region**	***r***	***p value***	***Cohen's d***
Perirhinal cortex	−0.65	0.0005	1.80
Olfactory bulb	0.52	0.0099	1.40
Olfactory nucleus	0.51	0.0101	2.72
Frontal pole	0.64	0.0008	3.18
Medial prefrontal cortex	−0.68	0.0003	-1.77
Dorsolateral prefrontal cortex	−0.42	0.0406	-2.72
Septal nucleus	−0.59	0.0025	-3.28
Auditory cortex	0.61	0.0016	2.29
Gustatory cortex	−0.42	0.0401	-2.08
S1	−0.64	0.0009	-4.84
Premotor	0.59	0.0022	2.39
M1	−0.74	<0.0001	-1.53
Intraparietal sulcus	0.72	<0.0001	3.05
V1	0.53	0.0082	1.71
V3	−0.56	0.0043	-2.80
Anterior cingulate	0.54	0.0059	1.87
Posterior cingulate	−0.57	0.0039	-2.13
Prostriate area	0.44	0.0301	0.73
Bed nucleus of the stria terminalis	0.49	0.0156	0.43
Claustrum	−0.64	0.0008	-1.45
Globus_pallidus	−0.81	<0.0001	-1.61
Hippocampal_formation	−0.67	0.0003	-1.88
Subiculum	−0.60	0.002	-0.77
Thalamus	−0.51	0.0104	-2.59

Supplementary Table 3. Significant correlation between cortical area volume and MD values and effect size

Region	*r*	*p value*	*Cohen's d*
Ventrolateral prefrontal cortex	0.45	0.0258	1.20
Inferior temporal area	0.48	0.0186	1.95
Ventral postal parietal area	0.72	<0.0001	5.72
V1	0.54	0.0065	4.05
Caudate nucleus	0.49	0.0154	1.75
Claustrum	0.49	0.0456	2.31
Medial geniculate nucleus	0.52	0.0087	2.67
Thalamus	0.46	0.025	1.97
Subthalamic nucleus	0.50	0.012	3.12

Supplementary Table 4. Significant correlation between cortical area volume and RD values and effect size

Region	*r*	*p value*	*Cohen's d*
Ventrolateral prefrontal cortex	0.41	0.045	1.13
M1	0.47	0.0206	0.98
Ventral postal parietal area	0.70	0.0001	4.28
Medial geniculate nucleus	0.45	0.0281	2.02
Thalamus	0.45	0.029	1.95
Subthalamic nucleus	0.44	0.0318	2.04

Supplementary Table 5. Significant correlation between cortical area volume and AD values and effect size

Region	*r*	*p*	*Cohen's d*
Olfactory bulb	0.66	0.0004	4.10
Olfactory_nucleus	0.75	<0.0001	4.04
Frontal_pole	0.80	<0.0001	4.16
Orbitofrontal_cortex	0.75	<0.0001	3.77
Medial_ventral_ prefrontal cortex	0.76	<0.0001	4.34
Medial_ prefrontal cortex	0.73	<0.0001	4.31
Ventrolateral_ prefrontal cortex	0.63	0.0009	3.97
Septal_nucleus	0.43	0.0352	4.06
Auditory_cortex	0.45	0.0283	4.00
S1	0.48	0.0164	3.49
S2	0.48	0.0169	6.33
Premotor	0.60	0.0018	2.41
M1	0.71	0.0001	3.09
Temporopolar_area	0.42	0.0397	4.38
Inferior_temporal_area	0.70	0.0002	2.24
Superior_temporal_polysensory_cortex	0.67	0.0004	3.60
Insular_cortex	0.44	0.031	3.16
Ventral_postal_parietal_area	0.78	<0.0001	6.69
Intraparietal sulcus	0.81	<0.0001	3.70
V1	0.61	0.0016	3.29
V2	0.54	0.0067	5.34
V3	0.50	0.0121	4.10
V6	0.55	0.0057	3.17
Anterior_cingulate	0.67	0.0004	2.60
Posterior_cingulate	0.50	0.0123	4.10
Retrosplenial_cortex	0.56	0.0042	2.49
Precuneus	0.61	0.0017	4.92
Prostriate_area	0.54	0.006	4.37
Accumbens_nucleus	0.64	0.0008	3.67
Parahippocampal_gyrus	0.60	0.002	3.91
Bed_nucleus_of_the_stria_terminalis	0.63	0.001	3.95
Caudate_nucleus	0.47	0.0196	3.41
Putamen	0.58	0.0031	4.52
Claustrum	0.58	0.003	2.70
Hippocampal_formation	0.49	0.0149	6.60
Subiculum	0.42	0.0415	9.80
Medial_geniculate_nucleus	0.69	0.0002	3.77
Dorsolateral_geniculate_nucleus	0.59	0.0022	4.62
Thalamus	0.73	<0.0001	4.52
Subthalamic_nucleus	0.62	0.0013	4.28

Supplementary Table 6. Significant correlations between in vivo and ex vivo differentiated brain area volumes and FA values

**Region**	***r***	***p value***
Ventrolateral prefrontal cortex	-0.80	0.002
Postal parietal area	0.65	0.021
Superior temporal rostral area	-0.62	0.029
Subiculum	-0.62	0.030
V3	-0.61	0.036
Perirhinal cortex	-0.59	0.044

Supplementary Table 7. Significant correlations between in vivo and ex vivo differentiated brain area volumes and MD values

**Region**	***r***	***p value***
V1	-0.41	0.011
**Superior colliculus**	-0.59	0.043
**Medial prefrontal cortex**	0.59	0.044
**Retrosplenial cortex**	-0.59	0.045
Premotor	-0.59	0.045
**Substantia nigra**	-0.59	0.045

Supplementary Table 8. Significant correlations between in vivo and ex vivo differentiated brain area volumes and RD values

**Region**	***r***	***p value***
**Retrosplenial cortex**	-0.75	0.005
**V1**	-0.72	0.009
**Insular cortex**	-0.64	0.024
**V2**	-0.61	0.034
**Premotor**	-0.60	0.040
**Entorhinal cortex**	-0.59	0.041

Supplementary Table 9. Significant correlations between in vivo and ex vivo differentiated brain area volumes and AD values

Region	*r*	*p value*
**Thalamus**	-0.65	0.023
**Dorsolateral prefrontal cortex**	-0.61	0.034
**Globus pallidus**	-0.58	0.048

Supplementary Table 10. Significant correlations between in vivo and ex vivo differentiated white matter volume and DTI indices

	**r**	**p**
**Mean diffusivity**	**0.14**	**0.66**
**Axial diffusivity**	**-0.39**	**0.203**
**Radial diffusivity**	**0.14**	**0.66**
**Fractional anisotropy**	**-0.55**	**0.065**

Supplementary Table 11. Brain structure atlas

Number of regions	Region name
1	Piriform cortex
2	Entorhinal cortex
3	Perirhinal cortex
4	Olfactory bulb
5	Olfactory nucleus
6	Frontal pole
7	Orbitofrontal cortex
8	Medial ventral prefrontal cortex
9	Medial prefrontal cortex
10	Dorsolateral prefrontal cortex
11	Ventrolateral prefrontal cortex
12	Premotor
13	primary motor cortex
14	primary somatosensory cortex
15	secondary somatosensory cortex
16	Ventral postal parietal area
17	intraparietal sulcus
18	Postal parietal area
19	Gustatory cortex
20	Temporopolar area
21	Inferior temporal area
22	Mid-temporal area
23	Superior temporal rostral area
24	Superior temporal polysensory cortex
25	Insular cortex
26	Auditory cortex
27	V1
28	V2
29	V3
30	V6
31	Septal nucleus
32	Anterior cingulate
33	Posterior cingulate
34	Retrosplenial cortex
35	Precuneus
36	Parahippocampal gyrus
37	Hippocampal formation
38	Subiculum
39	Amygdala
40	Prostriate area
41	Accumbens nucleus
42	Substantia nigra
43	Bed nucleus of the stria terminalis
44	Caudate nucleus
45	Putamen
46	Claustrum
47	Globus pallidus
48	Medial geniculate nucleus
49	Dorsolateral geniculate nucleus
50	Thalamus
51	Subthalamic nucleus
52	Superior colliculus

Supplementary Figure 1. Image showing brain regions whose volumes did not decrease significantly between *in vivo* and *ex vivo* conditions following perfusion fixation

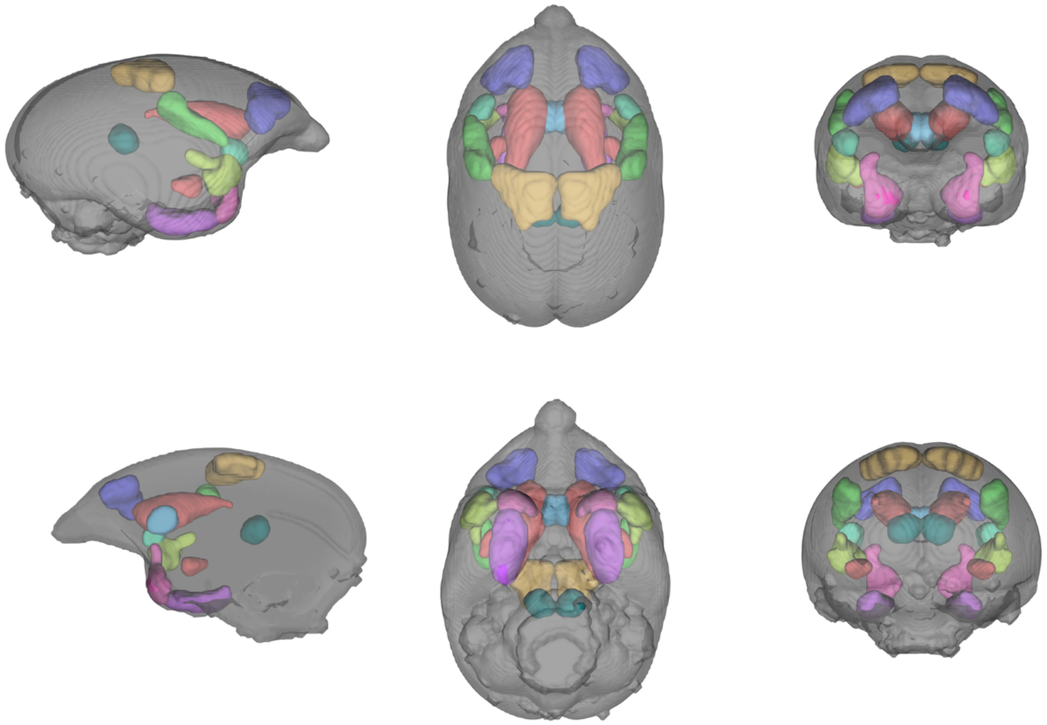

Ten regions that did not show statistically significant differences between *in vivo* and *ex vivo* brains are shown. These regions are the pisiform cortex, intraolfactory cortex, dorsolateral prefrontal cortex, septal nucleus, gustatory cortex, secondary somatosensory cortex, superior temporal rostrum, postal parietal region, caudate nucleus, and superior colliculus.

Supplementary Figure 2. Flowchart of the image registration and analysis process

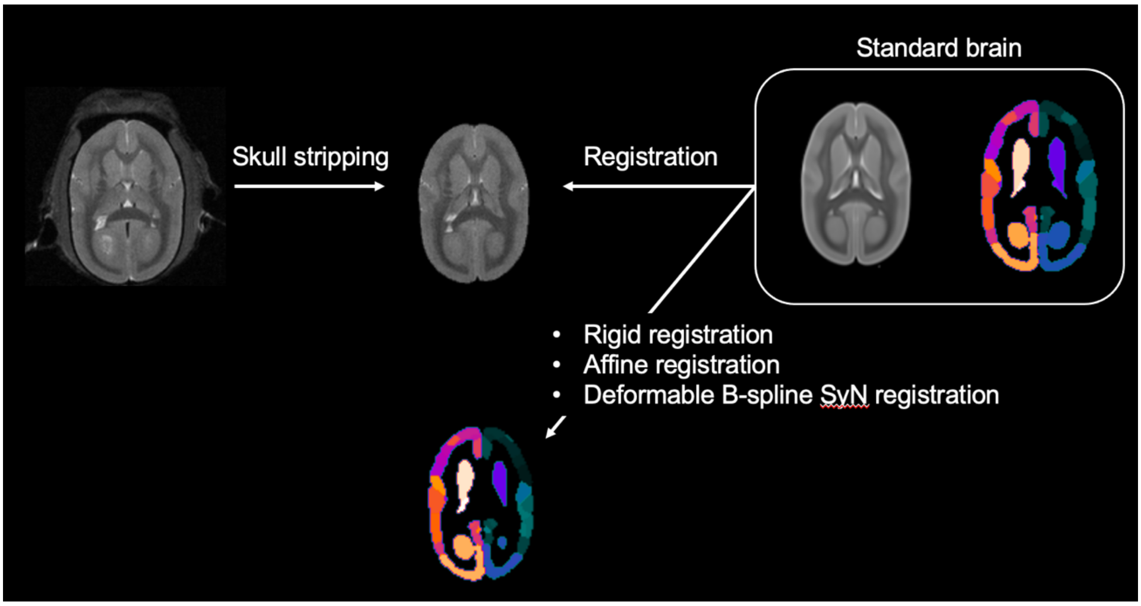

This diagram illustrates the step-by-step procedure from initial skull stripping to final volume comparisons between in vivo and ex vivo brain regions.

Supplementary Figure 3. Examples of the actual images acquired for T2 weighted image in in vivo

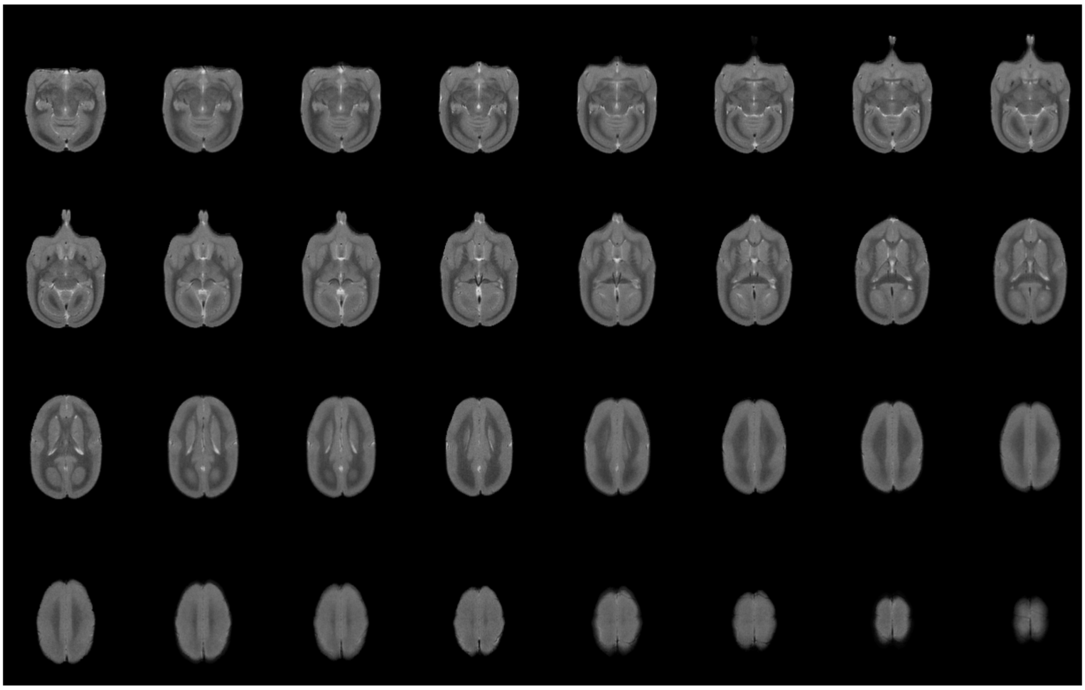

Examples of T2-weighted images of the in vivo brain under the imaging parameters used in this study are shown.

Supplementary Figure 4. Examples of the actual images acquired for T2 weighted image in ex vivo

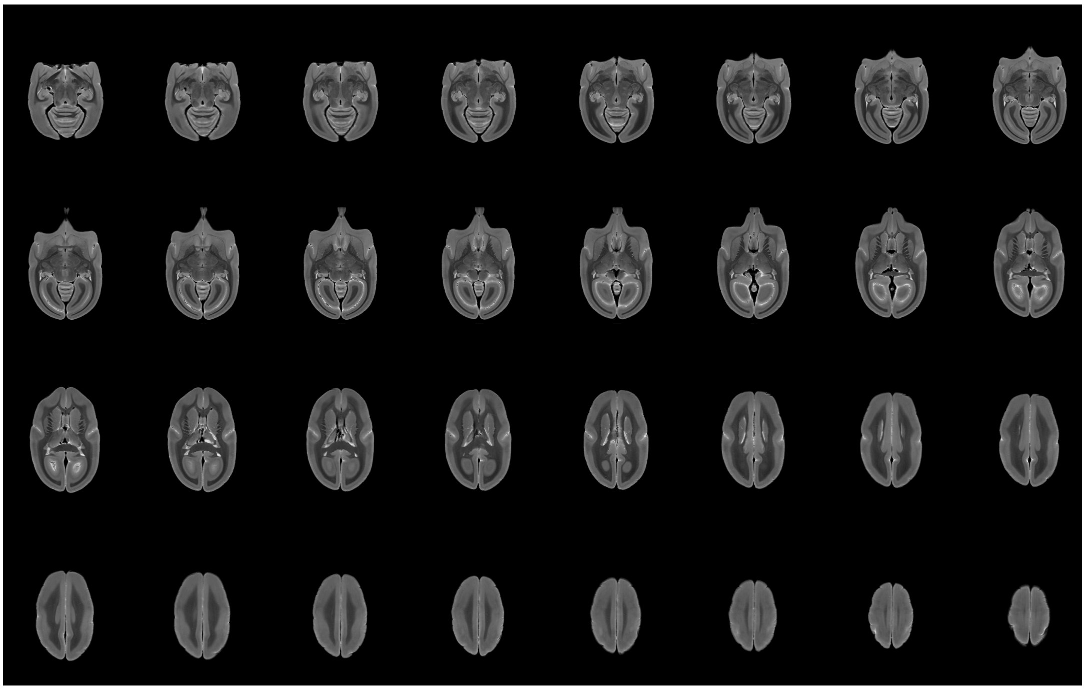

Examples of T2-weighted images of the ex vivo brain under the imaging parameters used in this study are shown.

Supplementary Figure 5. Sample of in vivo and ex vivo DWI images.

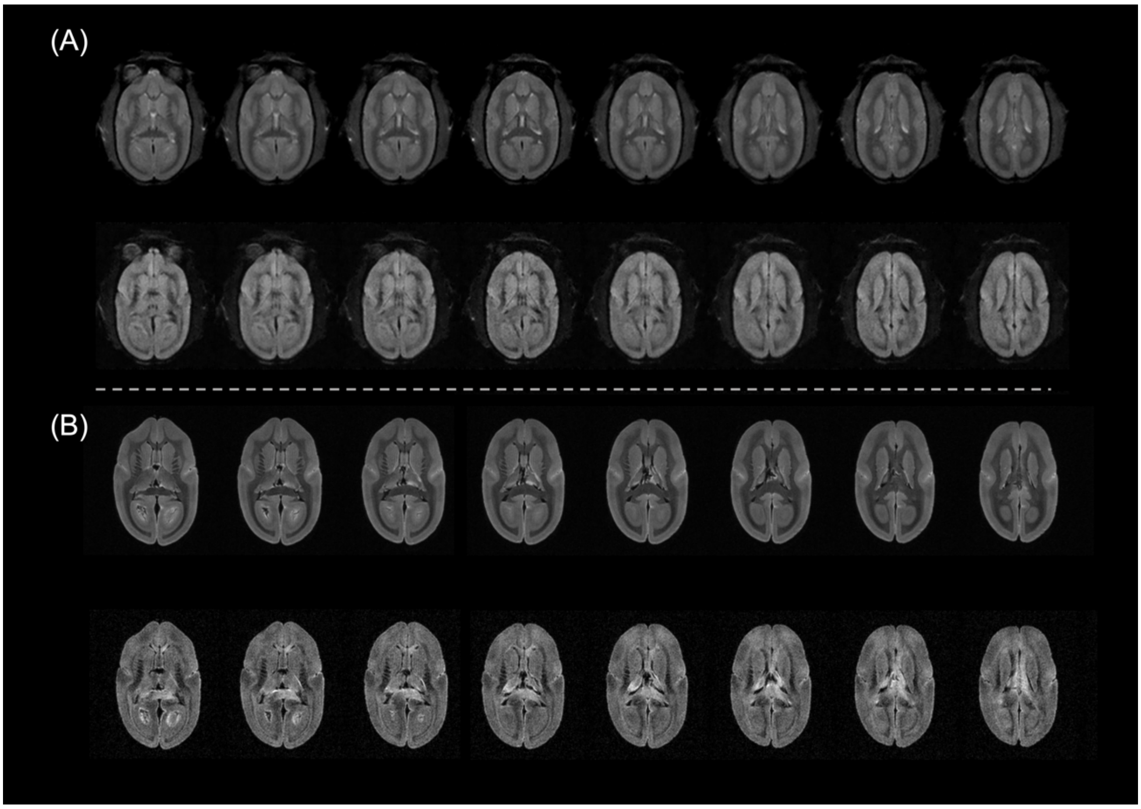

(A) represents in vivo DWI data, with the top row showing a b=0 image and the bottom row showing an image with a diffusion gradient applied (b=1000 s/mm²). (B) represents ex vivo DWI data, with the top row showing a b=0 image and the bottom row showing an image with a diffusion gradient applied (b=3000 s/mm²).

Supplementary Figure 6. DTI indices and track weighted images

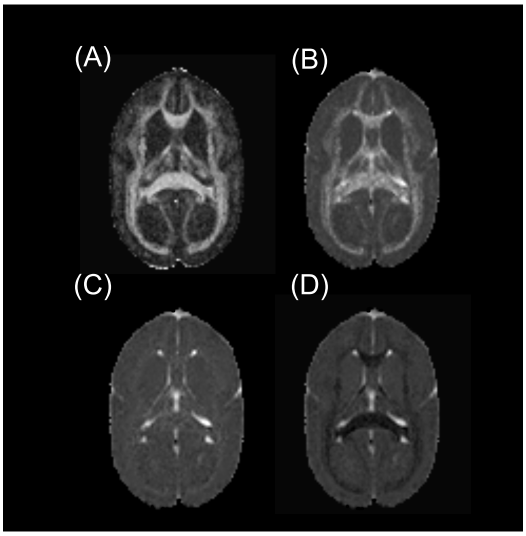

(A) represents Fractional Anisotropy (FA) images, (B) represents Axial Diffusivity (AD), (C) represents Mean Diffusivity (MD), and (D) represents Radial Diffusivity (RD) images.
